# Nontargeted Metabolomic Analysis of Four Different Parts of *Platycodon grandiflorum* Grown in Northeast China

**DOI:** 10.3390/molecules22081280

**Published:** 2017-08-03

**Authors:** Cuizhu Wang, Nanqi Zhang, Zhenzhou Wang, Zeng Qi, Hailin Zhu, Bingzhen Zheng, Pingya Li, Jinping Liu

**Affiliations:** 1 School of Pharmaceutical Sciences, Jilin University, Fujin Road 1266, Changchun 130021, China; wangcz15@mails.jlu.edu.cn (C.W.); zhangnq15@mails.jlu.edu.cn (N.Z.); wzz16@mails.jlu.edu.cn (Z.W.); qizeng15@mails.jlu.edu.cn (Z.Q.); zhuhl14@mails.jlu.edu.cn (H.Z.); zhengbz15@mails.jlu.edu.cn (B.Z.); 2 National and Local Joint Engineering Research Center for Ginseng Innovative Drugs Development, Western Chaoyang Road 45, Changchun 130021, China

**Keywords:** *Platycodon grandiflorum*, nontargeted metabolomic analysis, different part, UPLC-QTOF-MS^E^

## Abstract

Platycodonis radix is extensively used for treating cough, excessive phlegm, sore throat, bronchitis and asthma in the clinic. Meanwhile, the stems, leaves and seeds of *Platycodon grandiflorum* (PG) have some pharmaceutical activities such as anti-inflammation and anti-oxidation effects, etc. These effects must be caused by the different metabolites in various parts of herb. In order to profile the different parts of PG, the ultra-high performance liquid chromatography combined with quadrupole time-of- flight mass spectrometry (UPLC-QTOF-MS^E^) coupled with UNIFI platform and multivariate statistical analyses was used in this study. Consequently, for the constituent screening, 73, 42, 35, 44 compounds were characterized from the root, stem, leaf and seed, respectively. The stem, leaf and seed contain more flavonoids but few saponins that can be easily discriminated in the root. For the metabolomic analysis, 15, 5, 7, 11 robust biomarkers enabling the differentiation among root, stem, leaf and seed, were discovered. These biomarkers can be used for rapid identification of four different parts of PG grown in northeast China.

## 1. Introduction

It is well-known that there are both chemical and pharmacological differences in different parts of herbs. Taking *Aristolochia mollissima* Hance as an example, the fruits are used to treat cough and asthma, the roots have obvious antihypertensive effects, while the stems and leaves are rheumatoid medicines. This phenomenon also exists in other herbs, such as *Lycium barbarum, Polygonum Multiflorum* Thunb., *Trichosanthes kirilowii* Maxim, *Ephedra sinice* Stapf, etc. [1].

As both food and medicine, *Platycodon grandiflorum* (Jacq.) A. DC. (PG) is known as “Jiegeng” in China, “Huridunzhaga” in Mongolia, “Kikyo” in Japan and “Doraji” in North Korea [2]. In clinical, the root of PG which has various biological activities, such as apophlegmatic and antitussive [3], anti-inflammation [4], immunoregulation [5], anti-oxidant [6], etc., has been widely used for the treatment of cough, excessive phlegm, and sore throat. In addition, the stem and leaf of PG also have anti-inflammatory [7] and anti-oxidant [8,9] activities, while research on the pharmacological effects of PG seed is currently non-existent.

PG is a rich source of different natural products with various structural patterns. Around 100 compounds have been isolated from the roots of PG, including steroidal saponins, flavonoids, phenolic acids, polyacetylenes, sterols, etc. [2]. Triterpenoid saponins, mainly of the oleanane family pentacyclic type, are the active components of the root of PG [10]. Several flavonoids and phenolic acids were isolated from the aerial parts of PG [11]. Two glycosides and four flavonoids were isolated from the seeds of PG [12]. Recently, instead of traditional separation and identification method, a combination of ultra-high performance liquid chromatography (UHPLC) separation, quadrupole time-of-flight tandem mass spectrometry (QTOF-MS/MS) detection and automated data processing software UNIFI with scientific library was innovatively used for screening and identifying chemical components in herbal medicines [13,14] and traditional Chinese medicine formulas [15]. In 2015, Lee et al. reported the global profiling of various metabolites in PG by UPLC-QTOF/MS [16]. In that paper, a total of 20 metabolites were characterized from the roots, and 56 compounds from stems and leaves of PG grown in Korea. Herbs collected from different regions will show certain differences both in chemical constituents and in pharmacological activities [17]. For example, saponins in the root of PG from different sites in Gyeongnam Province, Korea showed different contents [18]. The ^1^H-NMR-based metabolomics with OPLS-DA statistical models was used to cluster the ginseng samples from Korea and China, and the result suggested that the chemical profiles from two countries are quite different due to their different geographical origins [19]. Hence, in order to illustrate different chemical constituents from the different regions and from the different parts of the plants, and to better clarify the pharmacological fundamental substances of PG, the root, stem, leaf and seed of PG produced in Jilin Province, China were taken as samples in this paper.

Metabolomics, including targeted and untargeted complementary approaches, is primarily concerned with identification and quantitation of small-molecule metabolites (<1500 Da) [20]. Recently, because of its ability to profile diverse classes of metabolites, untargeted metabolomics has been widely used to compare the overall metabolic composition of different samples [21]. An untargeted analysis approach is mainly applied in metabolite identification through mass-based search followed by manual verification [20] Being a sensitive, efficient, reliable, accurate and nondestructive method, UPLC-QTOF-MS has been widely used recently in this kind of analysis, such as exploring the early detection of mycotoxins in wheat [22], estimating compliance to a dietary pattern [23], exploring the bioavailability of the secoiridoids from a seed/fruit extract in human healthy volunteers [24], evaluating the enantioselective metabolic perturbations in MCF-7 cells after treatment with *R*-metalaxyl and *S*-metalaxyl [25].

In this study we focus on both the quickly chemical components’ screening and the non-targeted metabolomic analysis of the root, stem, leaf and seed of PG. UPLC-QTOF-MS^E^, UNIFI platform and multivariate statistical analyses, such as principal component analysis (PCA) and orthogonal partial least squares discriminant analysis (OPLS-DA) were used to profile the four different plant parts and to find the biomarkers among these four parts of PG grown in northeast China. 

## 2. Results

### 2.1. Identification of Components from Different Parts of PG

**Figure 1:**
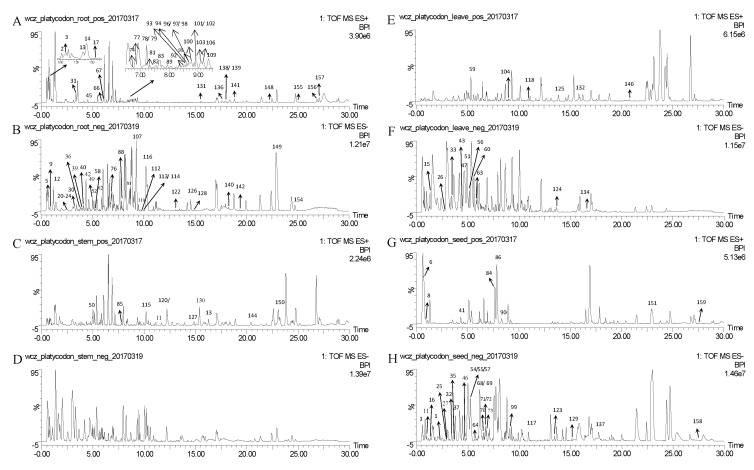
The representative base peak intensity (BPI) chromatograms of root in positive (**A**) and negative (**B**) modes; of stem in positive (**C**) and negative (**D**) modes; of leaf in positive (**E**) and negative (**F**) modes; of seed in positive (**G**) and negative (**H**) modes.

**Figure 2:**
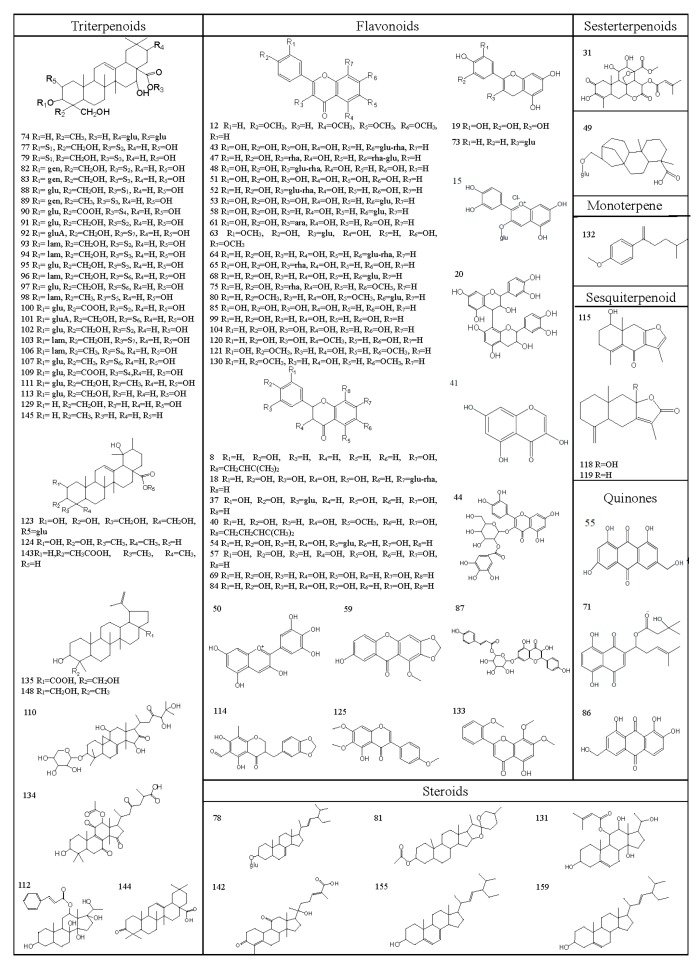

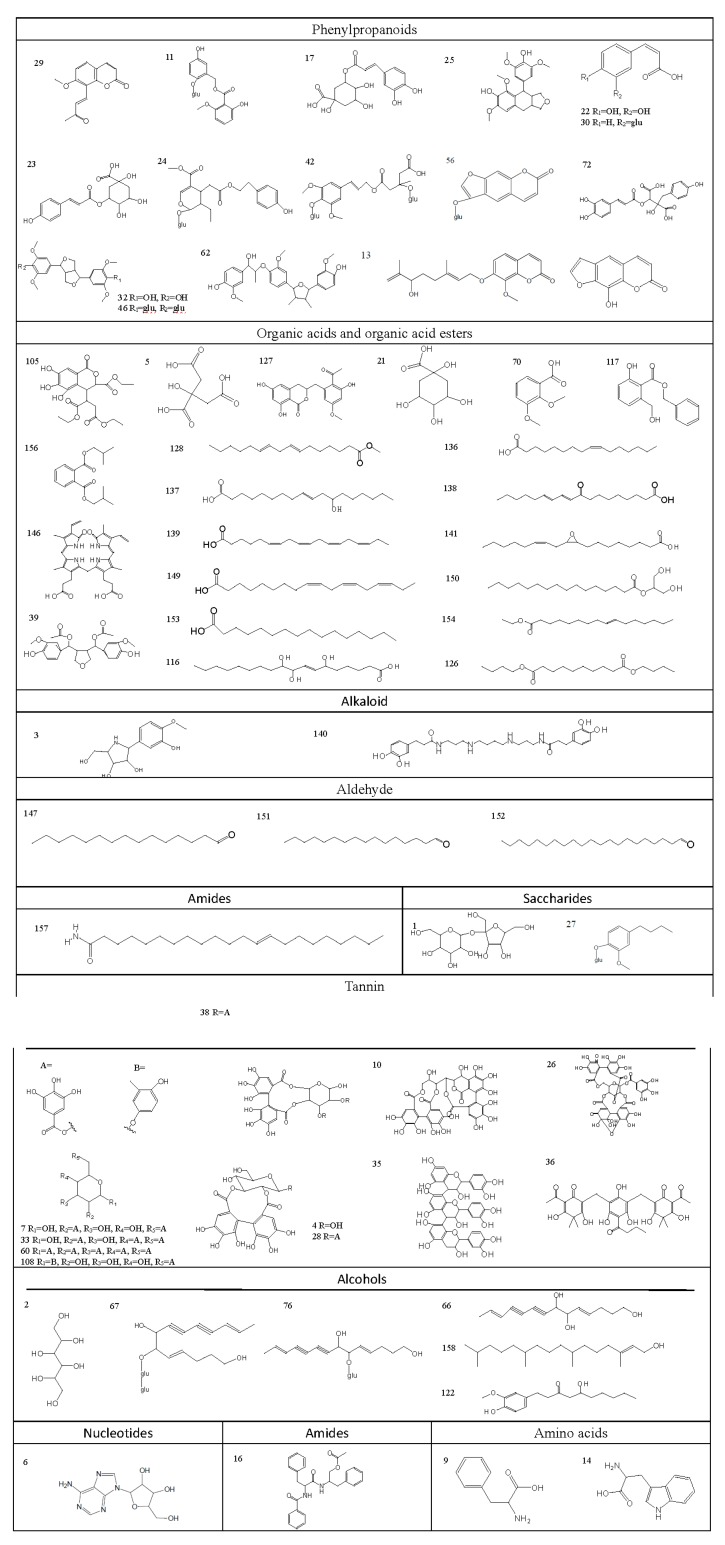
Chemical structures of compounds identified in PG.

As a result, a total of 159 compounds were identified or tentatively characterized in both positive and negative mode from the four parts of PG, the base peak intensity (BPI) chromatograms are shown in Figure 1, and their chemical structures are shown in Figure 2. More specifically, 73, 42, 35, 44 compounds were characterized from the root, stem, leaf and seed respectively (Table 1), including triterpenoid saponins, organic acids, steroids, phenols, flavonoids, alcohols, amino acids, coumarins, terpenoids, alkaloids and amides and so on. 

For the compounds which have isomers, they may be distinguished by their characteristic MS fragmentation patterns reported in literature, or may be compared with the retention times of reference standards. Taking compounds 98 and 106 as example, both have the same protonated ion [M + H]^+^ at *m*/*z* 1413.6530 and 1413.6530. In the results, they matched 3″-*O*-acetylpolygalacin D2 and 2″-*O*-acetylpolygalacin D2, respectively.

Their identical MS fragment pattern were similar. But according to the literature, the C3-glucoside was eluted earlier than the C2-glucoside [26,27,28] in the ESI-BPI chromatogram, so the compound with the earlier RT was identified as the C3-glucoside, 3″-*O*-acetylpolygalacin D2, and the other one with the later RT was identified as the C2-glucoside, 2″-*O*-acetylpolygalacin D2.

### 2.2. Biomarker Discovery for Differentiating Four Parts of PG

**Figure 3:**
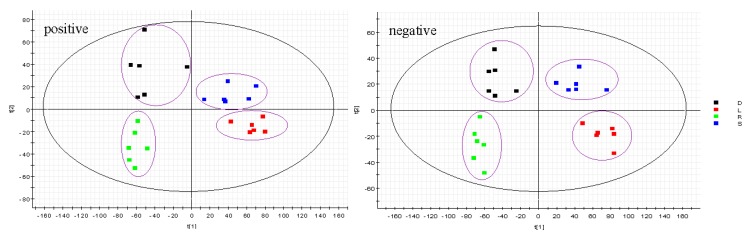
PCA of root (R), stem (S), leaf (L) and seed (D) of PG in positive mode and negative mode.

The PCA 2D plots of the samples from the root, stem, leaf and seed groups were classified in four clusters according to their common spectral characteristics (Figure 3). That means the four parts of PG could be easily differentiated.

**Figure 4:**
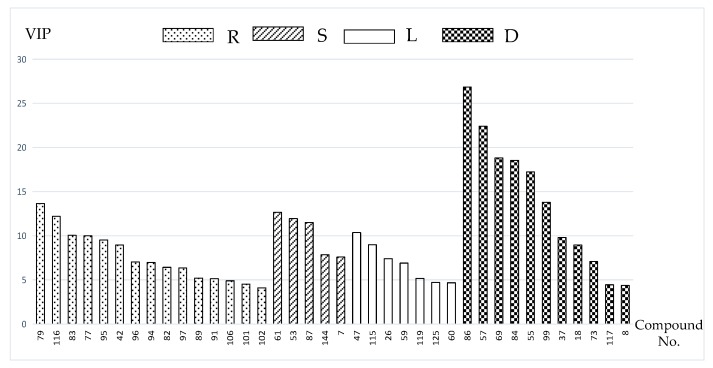
VIP value obtained from OPLS-DA model of the potential markers in root (R), stem (S), leaf (L) and seed (D) of PG.

**Figure 5:**
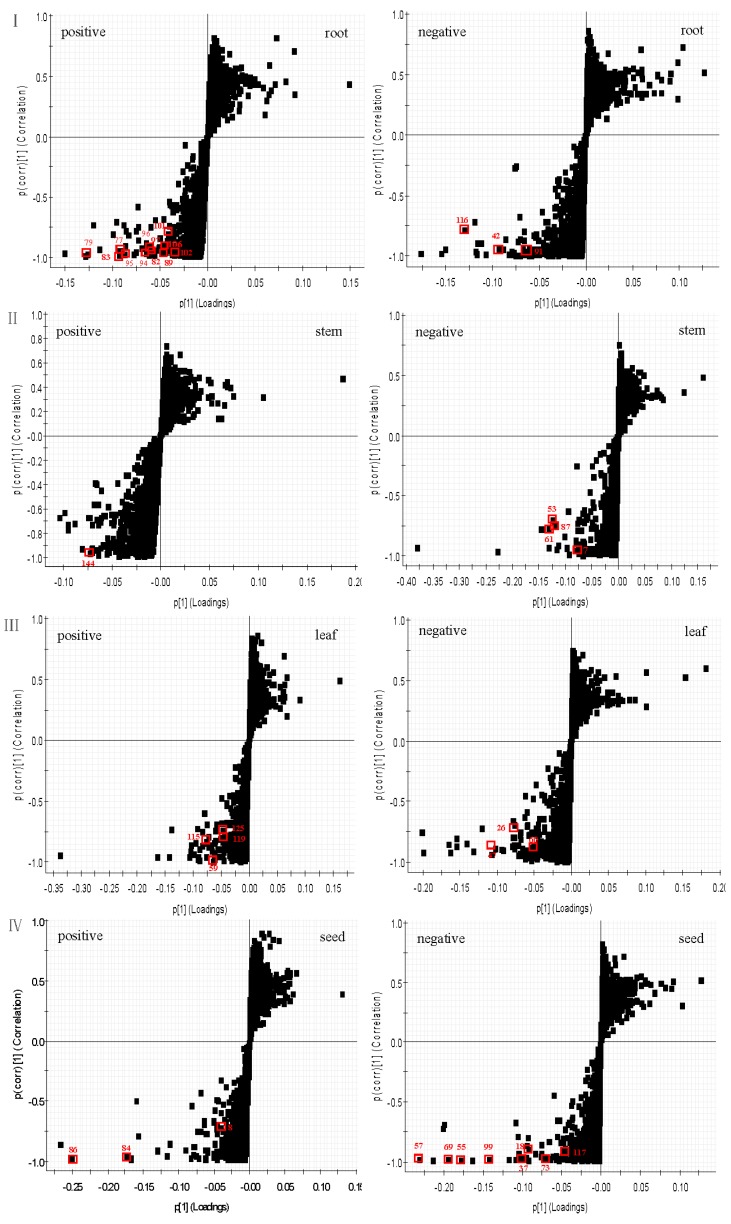
The OPLS-DA/S-plots of root (I), stem (II), leaf (III) and seed (IV) of PG in positive mode and negative mode.

**Figure 6:**
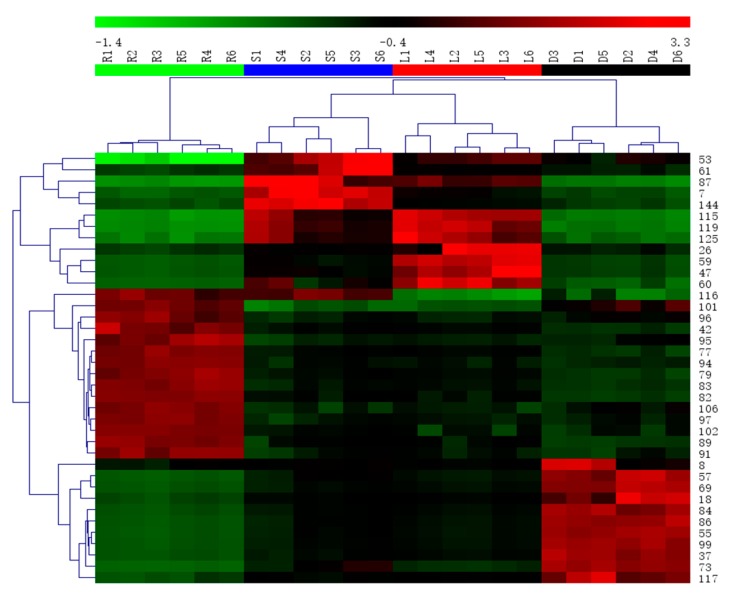
Heatmap visualizing the intensities of potential biomarkers.

In order to differentiate one part from other three parts, the OPLS-DA models were built in both positive and negative modes. Then, OPLS-DA score plot, S-plot, variable trend and VIP (variable importance in the projection) values were obtained to understand which variables are the responsible for this sample separation [29]. Based on VIP values (VIP > 4) (Figure 4) and *p* values (*p* < 0.05) [30] from univariate statistical analysis, 38 robust known biomarkers enabling the differentiation among root, stem, leaf and seed, were discovered and marked in S-plots (Figure 5). In order to systematically evaluate the biomarkers, a heatmap was generated from these biomarkers (shown in Figure 6), which shows distinct segregation among the four parts.

## 3. Discussion

There are 73, 42, 35, 44 compounds that were characterized from the root, stem, leaf and seed, respectively. As the results show, 95 compounds were identified in ESI(−) mode and 64 compounds were identified in ESI(+) mode. According to the BPI chromatograms of the four parts of PG, it seems that ESI(−) ionization mode is better than ESI(+) based on the quantity and the responses of the identified compounds, but it is still necessary to run the ESI(+) mode because some compounds showed better respond than in ESI(−) mode.

Compared with the results from previous studies [2,8,16,31,32], 56 chemical components were identified for the first time in Campanulaceae. The stem, leaf and seed contain more flavonoids but few saponins that can be easily discriminated from the root. In previous study, various metabolites in Korean *Platycodon grandiflorum* were profiled by UPLC-QTOF/MS [16]. Compared with the root of PG in Korea, there were only nine constituents (compounds **5**, **31**, **76**, **79**, **83**, **91**, **94**, **95**, **97**) in common. Meanwhile, the stems and leaves of PG in Korea and in China are both rich in natural components with various structural patterns, including triterpenoid saponins, flavonoids, organic acids, phenols, alcohols, amino acids, coumarins and amino acids, etc., but there are only two similar chemical components (compounds **99**, **104**). It is also interesting that there are eleven components (compounds **5**, **14**, **17**, **21**, **23**, **31**, **52**, **83**, **94**, **95**, **97**) reported in stems and leaves of PG in Korea that were found in the root of PG in China. The reason for this phenomenon may be the different analytical methods and the different growing locations.

In this paper, 38 robust known biomarkers enabling the differentiation among root, stem, leaf and seed, were discovered. For the root part, there are 15 potential biomarkers including triterpenoid saponins (**77**, **79**, **82**, **83**, **89**, **91**, **94**, **95**, **96**, **97**, **101**, **102**, **106**), an organic acid (**116**) and a phenyl-propanoid (**42**). For stem part, there are five potential biomarkers including flavonoids (**53**, **61**, **87**), a tannin (**7**) and a triterpenoid saponin (**144**). For leaf part, there are seven potential biomarkers including flavonoids (**47**, **59**, **125**), sesquiterpenoids (**115**, **119**) and tannins (**26**, **60**). For seed part, there are 11 potential biomarkers including flavonoids (**8**, **18**, **37**, **57**, **69**, **73**, **84**, **99**), quinones (**55**, **86**) and an organic acid (**117**). These robust biomarkers enabling the differentiation among root, stem, leaf and seed can be used for rapid identification of four different parts of PG grown in northeast China.

Even so, there are still some unresolved issues. Firstly, pharmaceutical effects associated with these robust biomarkers or these identified compounds should be screened in the future. Additionally, as shown in BPI chromatograms, though 159 compounds were identified there are still many unidentified components. Further research should be carried on based on the formula of these unknown compounds [13]. Most importantly, the stems and leaves of PG should be developed and utilized due to the presence of so many different components from the root. This comprehensive and unique phytochemical profile study revealed the structural diversity of secondary metabolites and the different patterns in various parts of PG. The method developed in this study can be used as a standard protocol for discriminating and predicting parts of PG directly.

## 4. Experimental Section

### 4.1. Materials and Reagents

All samples were harvested from Jilin Province, China, as listed in Table 2, and identified by Professor Ping-Ya Li (School of Pharmaceutical Sciences, Jilin University, Changchun, China). The voucher specimens (No. 2016121-2016144) had been deposited at the Research Center of Natural Drug, School of Pharmaceutical Sciences, Jilin University, Changchun, China. The cultivation ages of the roots are all 2 years, while the others are all 1 year old.

Acetonitrile and methanol suitable for UHPLC-MS purchased from Fisher Chemical Company (Geel, Belgium). Formic acid for UPLC was purchased from Sigma-Aldrich (St. Louis, MO, USA). Deionized water was purified using a Millipore water purification system (Millipore, Billerica, MA, USA). All other chemicals were of analytical grade. Fourteen standard compounds including platycodin D (111851-201607), mannitol (100533-201304), citric acid (111679-201602), phenylalanine (140676-201405), tryptophan (140686-201303), chlorogenic acid (110753-201716), caffeic acid (110885-201102), dibutyl sebacate (190102-201501), linolenic acid (111631-201605), sucrose (111507-201303), adenosine (110879-201202), monopalmitin (190011-201302), rutin (100080-201610), quercetin (100081-201610), were purchased from the National Institutes for Food and Drug Control (Beijing, China). Seven standard compounds including gallocatechin (201512013), quinine acid (20150321), brusatol (20150410), stigmasterol (20150111), xanthotoxol (20109376), delphinidin (20159567), and atractylenolide ІІІ (2014712) were purchased from Beijing Putian Genesis Biotechnology Co., Ltd. (Beijing, China). Nine standard compounds including deapioplatycoside E (160712), deapioplatycodin D (160518), -D_2_ (160407), platycoside E (160112), platycodin D_2_ (160721), -D_3_ (160909), platycoside G_3_ (160921), 2′-*O*-acetyl-platycodin D_2_ (160112), 3′-*O*-acetylplatycodin D_2_ (160923) were provided by Institute of Frontier Medical Science of Jilin University (Changchun, China). 

### 4.2. Sample Preparation and Extraction

The roots, stems, leaves and seeds of PG from the different sites were respectively air dried, ground and sieved (40 mesh) to give a homogeneous powder. Then 200 mg of the powder was respectively extracted thrice with 80% methanol at 80 °C for 3 h each time. After filtering, the extracts were combined, concentrated and evaporated to dryness. Finally, the desiccated extracts were dissolved and diluted with 80% methanol to 10.0 mL. The solution was filtered through a syringe filter (0.22 µm) and injected directly into the UPLC system. The volume injected was 2 μL for each run.

### 4.3. UPLC-QTOF-MSE

The UPLC analysis was performed by a Waters ACQUITY UPLC System. The column used was an ACQUITY UPLC BEH C18 (100 mm × 2.1 mm, 1.7 μm) from Waters Corporation (Milford, MA, USA). The mobile phases consisted of eluent A (0.1% formic acid in water, *v*/*v*) and eluent B (0.1% formic acid in acetonitrile, *v*/*v*) with flow rate of 0.4 mL/min with a liner gradient program: 10% B from 0 to 2 min, 10–90% B from 2 to 26 min, 90% B from 26 to 28 min, 90–10% B from 28 to 28.1 min, 10% B from 28.1 to 30 min. The temperature of the UPLC column and autosampler were set at 30 °C and 15 °C. Mixtures of 10/90 and 90/10 water/acetonitrile were used as the strong wash and the weak wash solvent respectively. 

The MS experiments were performed on a Waters Xevo G2-S QTOF mass spectrometer (Waters Co., Milford, MA, USA.) connected to the UPLC system through an electrospray ionization (ESI) interface. The optimized instrumental parameters were as follows: capillary voltage floating at 2.6 kV (ESI+) or 2.2 kV (ESI−); cone voltage at 40 V; source temperature at 120 °C, desolvation temperature at 300 °C and cone gas flow was 50 L/h, desolvation gas flow was 800 L/h. In MSE mode, collision energy of low energy function was set at 6 V, while ramp collision energy of high energy function was set at 20–40 V. To ensure mass accuracy and reproducibility, the mass spectrometer was calibrated over a range of 100–1600 Da with sodium formate. Leucine-enkephalin (*m*/*z* 556.2771 in positive ion mode; m/z 554.2615 in negtive ion mode) was used as the lockmass at a concentration of 200 ng/mL and flow rate of 20 μL/min. Data were collected in continuum mode, all the acquisition of data were controlled by the Waters MassLynx v.4.1 software ( waters, Milford, MA, USA).

### 4.4. Data Analysis

For the screening analysis, the raw data were processed using the streamlined workflow of UNIFI 1.7.0 software (Waters, Manchester, UK) to quickly identify the chemical components [15]. Besides the Waters Traditional Medicine Library in the UNIFI software, a self-built database was created including the information of chemical components from PG based on the literature and on-line databases such as China Full-text Journals Database (CNKI), PubMed, Medline, Web of Science and ChemSpider. Minimum peak area of 200 was set for 2D peak detection.The peak intensity of high energy over 200 counts and over 1000 counts for low energy were the selected parameters in 3D peak detection. A margin of error up to 5 ppm for identified compounds was allowed. Positive adducts containing +H, +Na, and negative adducts including +COOH and −H were selected. The verification of compounds was carried out by comparison with retention time of reference standards and characteristic MS fragmentation patterns reported in literature.

For metabonomics analysis, the raw data were processed by MarkerLynx XS V4.1 software for alignment, deconvolution, data reduction, etc. [33]. As a result, the list of mass and retention time pairs with corresponding intensities for all the detected peaks from each data file. The main parameters were as follows: retention time range 0–28 min, mass range 100–1600 Da, mass tolerance 0.10, minimum intensity 5%, marker intensity threshold 2000 counts, mass window 0.10, retention time window 0.20, and noise elimination level 6. The resulting data were analyzed by principle component analysis (PCA) and orthogonal projections to latent structures discriminant analysis (OPLS-DA). S-plots and VIP-plots were obtained via OPLS-DA analysis to find potential biomarkers that significantly contributed to the difference among the groups.

## 5. Conclusions

In the present study, UPLC-QTOF-MSE coupled with UNIFI platform and precise multivariate statistical analyses was used to profile the four parts of PG. For the constituent screening under the optimized conditions, a total of 159 chemical compounds (73, 42, 35, 44 compounds characterized from root, stem, leaf and seed, respectively) were identified from PG. The results showed various structural patterns including triterpenoid saponins, organic acids, steroids, phenols, flavonoids, alcohols, amino acids, coumarins, terpenoids, alkaloids and amides. The stem, leaf and seed contain more flavonoids but few saponins that can be easily discriminated from the root.

For the metabolomic analysis, four parts of PG were successfully discriminated into four different clusters. A total of 38 robust biomarkers were discovered. That is to say, 15, 5, 7, and 11 robust biomarkers enabling the differentiation among root, stem, leaf and seed, were characterized. These biomarkers can be suitable for the simultaneous differentiation of four different parts of PG, which is reported for the first time. In a word, these results provided the reliable characterization profiles and the differentiate components among root, leaf, stem and seed of PG grown in northeast China. The method developed in this study can be used as a standard protocol for discriminating and predicting the different parts of PG directly.

## Figures and Tables

**Table 1:** Compounds identified from different parts of PG by UPLC-QTOF-MS^E^.

No.	*t*_R_ (min)	Formula	Experimental (Da)	Theoretical (Da)	Mass Error (ppm)	Adducts	MS^E^ Fragmentation	Component Name	Source
1 *	0.59	C_12_H_22_O_11_	342.1169	342.1162	2.04	−H	323.0984, 195.0510, **161.0465**	Sucrose	D
2 *	0.60	C_6_H_14_O_6_	182.0797	182.0790	3.04	+Na	205.0689, **152.0713**	Mannitol	R
3	0.67	C_12_H_17_NO_5_	255.1114	255.1107	2.91	+H	256.1114, **226.1074**, 122.0375	Radicamine A	R
4	0.68	C_20_H_18_O_14_	482.0682	482.0697	−2.95	−H	**343.0676**, 301.0007, 274.0119, 191.0554, 152.0124	2,3-(*S*)-Hexahydroxydiphenoyl-d-glucose ^a^	S, L
5 *	0.71	C_6_H_8_O_7_	192.0278	192.0270	3.93	−H	191.0205, **173.0077**, 111.0089	Citric acid	R
6 *	0.75	C_10_H_13_N_5_O_4_	267.0974	267.0968	2.23	+H	**218.1020**, 136.0634	Adenosine	D
7	0.82	C_20_H_20_O_14_	484.0857	484.0853	0.78	−H	313.0568, 183.0308, **169.0156**, 152.0123	2,6-Di-*O*-Galloyl-β-d-glucose ^a^	S, L
8	0.85	C_20_H_20_O_4_	324.1347	324.1362	−4.38	+H	**203.0708**, 175.0758, 164.0463, 149.0602, 103.0556	Isobavachin ^a^	D
9 *	0.86	C_9_H_11_NO_2_	165.0796	165.0790	3.98	−H	164.0724, 147.0456, 103.0549	Phenylalanine	R
10	0.95	C_34_H_24_O_22_	784.0751	784.0759	−1.05	−H	421.0417, **337.0214**, 249.0416, 182.0223, 168.0074, 149.9967	Casuariin ^a^	S
11	0.97	C_21_H_24_O_11_	452.1341	452.1319	4.86	−H	**299.0771**, 289.0737, 271.0611, 165.0206, 137.0257	Curculigoside B ^a^	D
12	1.02	C_19_H_18_O_6_	342.1089	342.1103	−4.14	−H	211.0628, 181.0506, **179.0349**, 161.0240, 151.0404	5,6,7,4′-Tetramethoxyflavone ^a^	R
13	1.24	C_20_H_24_O_5_	344.1609	344.1624	−3.98	+Na	222.0916, **194.0973**, 182.0611, 127.0394	Schininallylol ^a^	R
14 *	1.35	C_11_H_12_N_2_O_2_	204.0903	204.0899	2.29	+H	**188.0706**, 144.0808, 132.0813, 118.0661	Tryptophan	R
15	1.36	C_21_H_21_ClO_11_	484.0775	484.0772	0.45	−H	309.0630, 287.0594, 124.0163, **109.0291**	Cyanidin 3-glucoside ^a^	L
16	1.37	C_27_H_28_N_2_O_4_	444.2034	444.2049	−3.41	−H	235.1215, **175.0626**, 173.0464, 131.0364, 105.0356	Aurantiamide acetate ^a^	D
17 *	1.73	C_16_H_18_O_9_	354.0950	354.0951	−0.22	+H	192.0663, **163.0396**, 145.0294, 135.0452	Chlorogenic acid	R
18	2.15	C_27_H_32_O_16_	612.1712	612.1690	3.51	−H	593.1511, 461.1313, **303.0532**, 285.0428, 177.0209, 151.0052	(2*R*,3*R*)-Taxifolin7-*O*-α-l-rhamnopyranosyl-(1→6)-β-d-glucopyranoside	D
19	2.30	C_30_H_26_O_12_	578.1430	578.1424	0.98	−H	449.0876, 425.0875, 407.0777, **289.0718**, 125.0257	Procyanidin B_1_^a^	D
20 *	2.31	C_15_H_14_O_7_	306.0738	306.0740	−0.53	+HCOO	**179.0349**, 167.0343, 163.0406, 161.0241, 109.0315	Gallocatechin	R
21 *	2.34	C_7_H_12_O_6_	192.0637	192.0634	1.53	−H	173.0480, **127.0406**, 116.0514, 111.0456	Quinine acid	R
22 *	2.35	C_9_H_8_O_4_	180.0425	180.0423	1.60	−H	161.0241, 135.0451, **133.0297**, 109.0315, 108.0224	Caffeic acid	R
23	2.36	C_16_H_18_O_8_	338.0993	338.1002	−2.62	−H	**191.0567**, 177.0195, 161.0243, 119.0505, 105.0351	3-*O*-*trans*-Coumaroylquinic acid	R
24	2.70	C_25_H_34_O_12_	526.2045	526.2050	−1.04	−H	**363.1452**, 315.1244, 179.0713, 167.0711, 149.0612	LucidumosideA ^a^	R
25	2.78	C_22_H_26_O_7_	402.1670	402.1679	−1.88	+HCOO	327.0884, **303.0885**, 297.0421, 209.0844, 137.0256	Neociwujiaphenol ^a^	D
26	2.81	C_41_H_28_O_27_	952.0809	952.0818	−0.97	−H	605.0777, 479.0469, **481.0642**, 453.0677, 246.0169	Geraniin ^a^	L
27	2.98	C_17_H_26_O_7_	342.1678	342.1679	−0.01	+HCOO	281.0651, **163.1130**, 121.0300	Citrusin C	D
28	2.99	C_27_H_22_O_18_	634.0813	634.0806	1.16	−H	601.0460, **463.0518**, 419.0617, 301.0007, 291.0156, 275.0208	Sanguiin H-4 ^a^	S
29	3.05	C_14_H_12_O_4_	244.0745	244.0736	3.14	+HCOO	203.0721, **187.0402**, 161.0250, 123.0457, 109.0303	cis-Osthenone	D
30	3.24	C_15_H_18_O_8_	326.1003	326.1002	0.33	+HCOO	**162.0552**, 129.0199, 121.0304	4-*O*-β-d-glucopyranosyl-*trans*-cinnamic acid ^a^	R, D
31 *	3.28	C_26_H_32_O_11_	520.1968	520.1945	4.46	+H	443.0984, 341.1392, **163.075**	Brusatol	R
32	3.42	C_22_H_26_O_8_	418.1631	418.1628	0.72	−H	359.1465, 179.0726, 164.0477, 149.0251, **125.0254**	(+)-Syringaresinol	D
33	3.55	C_27_H_24_O_18_	636.0969	636.0963	0.99	−H	483.0791, **465.0679**, 331.0667, 313.0578, 169.0163	2,4,6-Tri-*O*-galloyl-β-d-glucose ^a^	S,L
34 *	3.65	C_11_H_6_O_4_	202.0260	202.0266	−2.32	+HCOO	**163.0419**, 149.0244, 134.0373, 133.0304,	Xanthotoxol	S, L
35	3.67	C_45_H_38_O_18_	866.2079	866.2058	2.37	−H	**575.1207**, 407.0781, 289.0730, 179.0356	Arecatannin A_1_^a^	D
36	3.76	C_32_H_36_O_12_	612.2223	612.2207	2.59	−H	562.1866, 518.1583, **210.0880**, 135.0462	Filixic acid ABA ^a^	R
37	3.78	C_21_H_22_O_12_	466.1128	466.1111	3.59	−H	**285.0428**, 177.0208, 165.0568, 151.0053, 137.0257, 124.0178	Taxifolin-3-*O*-glucoside ^a^	D
38	3.80	C_34_H_26_O_22_	786.0915	786.0916	−0.08	−H	615.0646, 597.0511, 445.0416, **301.0021**, 125.0258	Collinin ^a^	S
39	3.82	C_24_H_28_O_9_	460.1739	460.1733	1.24	−H	414.1699, 389.1244, 193.0528, **137.0261**, 125.0258	Sanjidin A ^a^	R
40	4.30	C_22_H_24_O_6_	384.1560	384.1573	−2.96	+HCOO	**325.1065**, 313.1078, 310.0838, 150.0322	Sophoflavescenol ^a^	R
41	4.33	C_9_H_6_O_5_	194.0211	194.0215	−2.35	+H	177.0183, **153.0178**, 138.0309, 127.0398	3,5,7-Trihydroxychromone	D
42	4.38	C_29_H_42_O_18_	678.2395	678.2371	3.54	−H	**497.1692**, 453.1789, 323.0997, 291.1258, 161.0471	TangshenosideI	R
43	4.48	C_27_H_30_O_16_	610.1554	610.1534	3.24	−H	**463.0844**, 313.0580, 265.0370, 190.9983, 151.0043	Quercetin-7-*O*-rutinoside	L
44	4.50	C_28_H_24_O_16_	616.1084	616.1064	3.27	−H	313.0580, 190.9983, 177.0206, **169.0158**, 151.0043	2′′-*O*-Galloylhyperoside ^a^	S, L
45	4.53	C_11_H_12_O_3_	192.0791	192.0786	2.21	+H	193.0863, 167.0703, **161.0603**	Myristicin	R
46	4.66	C_34_H_46_O_18_	742.2707	742.2684	2.90	+HCOO	579.2040, **417.1564**, 181.0520, 149.0248	Syringaresinol-di-*O*-β-d-glucoside ^a^	D
47	4.72	C_33_H_40_O_19_	740.2178	740.2164	1.92	−H	593.1506, 575.1401, 429.0824, 335.0414, **284.0336**	Grosvenorine ^a^	S, L
48 *	4.93	C_27_H_30_O_16_	610.1550	610.1534	2.59	−H	401.0912, **301.0365**, 299.0205, 247.0609	Rutin	S, L, D
49	4.94	C_26_H_42_O_8_	482.2874	482.2880	−1.13	+HCOO	**261.1352**,179.1074,149.0608, 125.0589	17-*O*-β-d-Glucopyra-nosyl-16β-H-ent-kauran-19-oicacid ^a^	R
50 *	4.96	C_15_H_10_O_7_	302.0427	302.0427	0.15	+H	161.0264, **123.0099**, 109.0306, 107.0153	Delphinidin	S, L
51	4.97	C_15_H_10_O_8_	318.0368	318.0376	−2.25	+HCOO	**300.0266**, 264.0562, 176.0132, 148.0176	Quercetagetin	L
52	5.12	C_27_H_30_O_15_	594.1609	594.1585	4.02	−H	**285.0403**, 161.0459, 151.0038, 135.0452	Kaempferol-3-*O*-neohesperidoside	R
53	5.14	C_21_H_20_O_12_	464.0945	464.0955	−2.16	−H	313.0549, **300.0266**, 284.0330, 151.0041	Quercimeritrin	S,L,D
54	5.17	C_21_H_22_O_11_	450.1178	450.1162	3.61	−H	193.0156, 179.0574, 175.0051, **151.0052**, 135.0468	Dihydrokaempferol-5-*O*-β-d-glucopyranoside	D
55	5.24	C_15_H_10_O_6_	286.0463	286.0477	−4.95	−H	256.0372, 177.0180, 164.0487, **150.0300**, 123.0439, 107.0134	ω-Hydroxyemodin ^a^	D
56	5.25	C_17_H_16_O_9_	364.0780	364.0794	−3.53	+HCOO	337.0566, 278.0432, **202.0248**, 185.0254, 149.0251	Bergaptol-*O*-β-d-glucopyranoside	L
57	5.26	C_15_H_12_O_7_	304.0568	304.0583	−4.92	−H	285.0366, 243.0329, 152.0099, **150.0300**, 125.0238	Dihydroquercetin	D
58	5.27	C_21_H_20_O_11_	448.1005	448.1006	−0.19	−H	**285.0406**, 283.0256, 179.0569	Luteolin-7-*O*-glucopyranoside	R,D
59	5.40	C_15_H_10_O_6_	286.0479	286.0477	0.70	+H	**149.0216**, 139.0371, 123.0433, 111.0439	7-Hydroxy-1-methoxy-2-methoxyxanthone ^a^	S, L
60	5.57	C_41_H_32_O_26_	940.1163	940.1182	−1.96	−H	**769.0887**, 617.0782, 313.0565, 291.0150, 169.0158	1,2,3,4,6-Penta-*O*-galloyl-β-d-glucopyranoside ^a^	S, L
61	5.72	C_20_H_18_O_11_	434.0853	434.0849	0.80	−H	**300.0301**, 195.0321, 151.0050, 109.0305	Quercetin-3-*O*-arabinoside	S
62	5.76	C_30_H_36_O_8_	524.2409	524.2410	−0.19	+HCOO	453.1908, 339.1256, **195.0667**, 165.0570	Saucerneol C ^a^	R
63	5.79	C_23_H_24_O_13_	508.1224	508.1217	1.36	−H	315.0519, **207.0291**, 193.0506, 151.0044, 137.0246	Limocitrin-3-*O*-β-d-glucopyranoside ^a^	L
64	5.83	C_27_H_30_O_14_	578.1637	578.1636	0.24	−H	**269.0475**, 227.0364, 177.0203, 151.0050, 119.0513	Apigenin-7-*O*-β-d-rutinoside	D
65	5.84	C_21_H_20_O_11_	448.1016	448.1006	2.36	−H	295.0843, **284.0340**, 179.0362, 151.0411, 123.0102	Quercetin-3-*O*-α-l-rhamnoside	S
66	5.86	C_14_H_18_O_3_	234.1243	234.1256	−4.58	+H	175.0746, **163.0746**, 133.0647, 119.0860, 111.0811	Lobetyol	R
67	6.02	C_26_H_38_O_13_	558.2326	558.2312	2.37	+Na	**217.1197**, 199.1096, 145.0642, 128.0613, 115.0541	Lobetyolinin	R
68	6.12	C_21_H_20_O_10_	432.1040	432.1056	−3.82	−H	**268.0367**, 227.0341, 177.0181, 151.0037, 124.0168	Cosmosiin	D
69	6.17	C_15_H_12_O_6_	288.0643	288.0634	3.23	−H	271.0623, 177.0181, **151.0037**, 133.0297, 125.0254, 107.0143	Dihydrokaempferol	D
70	6.32	C_9_H_10_O_4_	182.0584	182.0579	2.65	−H	166.0263, 151.0040, **135.0452**, 108.0226	2,6-Dimethoxy benzoic acid	D
71	6.59	C_21_H_24_O_7_	388.1509	388.1522	−2.96	+HCOO	358.1066, 301.0369, 243.0306, **231.0308**, 151.0047	β-Hydroxyisovalerylshikonin ^a^	D
72	6.61	C_20_H_18_O_10_	418.0892	418.0900	−1.74	+HCOO	358.1066, 243.0306, **231.0308**, 178.9997, 151.0047, 121.0304	Cimicifugic acid D ^a^	D
73	6.70	C_21_H_24_O_10_	436.1373	436.1369	0.76	−H	**273.0781**, 255.0666, 179.0358, 149.0248, 123.0457	Epiafzelechin-3-*O*-β-d-allopyranoside ^a^	D
74	6.75	C_42_H_68_O_16_	828.4491	828.4507	−1.99	+H	667.4052, 651.4104, 505.3529, **487.3428**, 469.3321, 421.3113	Platycosaponin A	R
75	6.79	C_22_H_22_O_10_	446.1231	446.1213	3.66	+HCOO	**285.0424**, 187.0053, 163.0414, 124.0179	Rhamnocitrin-3-*O*-rhamnoside ^a^	S
76	6.81	C_20_H_28_O_8_	396.1793	396.1784	2.03	+HCOO	**215.1094**, 185.0984, 159.0826, 143.0724, 125.0616	Lobetyolin	R
77 *	6.85	C_64_H_104_O_34_	1416.6388	1416.6409	−1.49	+H	811.4487, **763.42581**, 647.37911, 485.3261	Deapio platycoside E	R
78	6.93	C_35_H_58_O_6_	574.4227	574.4233	−1.03	+H	472.3166, **463.3096**, 378.2044, 302.1716	α-Spinasterol glucoside	R
79 *	6.98	C_69_H_112_O_38_	1548.6799	1548.6832	−2.13	+H	1007.5104, 845.4571, **683.4034**, 521.3493, 485.3282	Platycoside E	R
80	6.99	C_23_H_24_O_11_	476.1314	476.1319	−0.84	+HCOO	433.1097, **345.0819**, 313.0554, 183.0309, 151.0041	5-Hydroxy-6,4′-dimethoxy-flavone-7-*O*-β-d-gluco-pyranoside	S
81	7.35	C_29_H_46_O_4_	458.3396	458.3396	−0.05	+H	**341.2455**, 217.1953, 149.1333, 121.1027	Neotigogenin acetate ^a^	R
82	7.57	C_58_H_94_O_29_	1254.5905	1254.5881	1.95	+H	931.4894, 845.4518, **799.4485**, 295.1007	Deapioplatycodin D_3_	R
83 *	7.68	C_63_H_102_O_33_	1386.6326	1386.6303	1.65	+H	1255.5937, 931.4894, 845.4518, **799.4484**, 665.3879, 441.1585	Platycodin D_3_	R
84	7.69	C_15_H_12_O_6_	288.0629	288.0634	−1.59	+H	255.0652, 179.0353, 163.0400, **153.0196**, 145.0295	3-Hydroxynaringenin ^a^	D
85 *	7.77	C_15_H_10_O_7_	302.0422	302.0427	−1.40	+H	243.0319, **151.0055,** 125.0260, 107.0157	Quercetin	S, L
86	7.86	C_15_H_10_O_6_	286.0488	286.0477	3.61	+H	269.0460, 257.0450, **241.0490**, 161.0239, 135.0453	6-Hydroxyaloeemodin ^a^	D
87	7.91	C_30_H_26_O_13_	594.1373	594.1373	−0.14	−H	447.0966, 429.0832, **285.0440**, 145.0316, 119.0513	Buddlenoid A ^a^	S, L
88	7.92	C_47_H_76_O_20_	960.4934	960.4930	0.39	+HCOO	869.4537, **715.3371**, 529.2698, 295.2034	Platycoside F	R
89 *	7.94	C_63_H_102_O_32_	1370.6373	1370.6354	1.40	+H	827.4398, 783.4476, 637.3944, 459.3430, 409.3090, **325.1130**	Platycoside G_3_	R
90	8.33	C_57_H_90_O_29_	1238.5577	1238.5568	0.71	+H	1107.5237, 957.4692, 895.4676, 811.4125, **697.3760**, 661.3582, 485.3245, 409.3094	Platyconic acid A	D
91 *	8.46	C_52_H_84_O_24_	1092.5397	1092.5353	4.07	−H	959.4846, 941.4753, **681.3871**, 663.3768, 649.3607, 503.3364, 485.3366, 295.1038, 277.0942	Deapioplatycodin D	R
92	8.48	C_59_H_92_O_30_	1280.5649	1280.5673	−1.90	+H	1017.4875, 999.4760, 931.4860, 829.4192, **697.3796**, 679.3651, 651.3761, 519.3316, 503.3334, 487.3377	Platycodin L	R
93 *	8.51	C_58_H_94_O_29_	1254.5847	1254.5881	−2.65	+H	931.4894, 845.4518, **799.4485**, 483.3065, 457.1533, 427.1433, 325.1116, 295.1007	Deapioplatycodin D_2_	R
94 *	8.62	C_63_H_102_O_33_	1386.6300	1386.6303	−0.26	+H	977.4981, 845.4558, 829.4604, 683.4031, 667.4073, 653.3919, **521.3488**, 485.3273	Platycodin D_2_	R
95 *	8.68	C_57_H_92_O_28_	1224.5778	1224.5775	0.23	+H	799.4485, 683.3961, 667.4052, **521.3444**, 503.3364, 485.3257	Platycodin D	R, D
96 *	8.73	C_65_H_104_O_34_	1428.6407	1428.6409	−0.15	+H	1297.6065, 1165.5621, 845.4520, 841.4580, 681.3837, 665.3903, **653.3884**, 617.3663, 519.3298, 485.3243	2′-O-Acetylplatycodin D_2_	R, D
97	8.78	C_59_H_94_O_29_	1266.5869	1266.5881	−0.93	+H	1003.5108, 841.4569, 823.4458, **683.3979**, 189.0749, 171.0641	Platycodin A	R, D
98	8.80	C_65_H_104_O_33_	1412.6458	1412.6460	−0.16	+H	985.4990, 823.4461, 635.3794, 617.3695, **503.3369**, 453.1605, 321.1182, 303.1076, 189.5707	3′′-*O*-Acetylpolygalacin D_2_	R
99	8.86	C_15_H_10_O_5_	270.0539	270.0528	4.15	−H	**151.0043**, 123.0099, 117.0359, 107.0154	Apigenol	D
100	8.87	C_52_H_82_O_25_	1106.5163	1106.5145	1.57	+H	975.4806, 931.4908, 829.4243, 811.4113, **697.3814**, 679.3695, 517.3151, 503.3373, 455.3161	Platyconic acid C	R
101	8.94	C_59_H_92_O_30_	1280.5705	1280.5673	2.47	+H	1017.4875, **829.4192**, 697.3796, 637.3939, 519.3316, 321.1178	Platycodin K	R, D
102	9.04	C_54_H_86_O_25_	1134.5444	1134.5458	−1.23	+H	1003.5108, 841.4569, 823.4458, **683.3979**, 321.1160, 189.0749	Platycoside B	R
103 *	9.10	C_65_H_104_O_34_	1428.6370	1428.6409	−2.71	+H	1297.6065, 955.4894, 841.4580, 813.4279, 797.4332, 681.3837, 665.3903, **653.3884**, 635.3780	3′-*O*-acetyl-platycodin D_2_	R
104	9.11	C_15_H_10_O_6_	286.0483	286.0477	1.85	+H	231.0662, 229.0504, 195.0289, **153.0187**,	Kaempferol	L
105	9.14	C_20_H_24_O_11_	440.1314	440.1319	−1.01	−H	393.0860, 303.0523, 257.0104, 231.0303, 177.0204	(-)-Chebulic acid triethyl ester ^a^	S, L
106	9.18	C_65_H_104_O_33_	1412.6430	1412.6460	−2.14	+H	823.4461, 503.3369, **485.3255**, 455.3156, 321.1182, 189.0757	2′′-*O*-acetylpolygalacin D_2_	R, D
107	9.23	C_59_H_94_O_28_	1250.5904	1250.5932	−2.23	−H	**1208.5857**, 1159.5571, 635.3812, 499.3046, 131.0337	2′-*O*-acetyl Polygalacin D	R
108	9.32	C_20_H_22_O_11_	438.1170	438.1162	1.78	−H	**419.0956**, 235.0654, 163.0050	6′-*O*-Galloyl-homoarbutin ^a^	S, L
109	9.37	C_54_H_84_O_26_	1148.5293	1148.5251	3.63	+H	1017.4908, 999.4786, 535.3279, 631.3477, **517.3170**, 499.3050, 453.3001, 321.1190, 189.0764	Platyconic acid D	R
110	9.45	C_35_H_54_O_11_	650.3666	650.3666	0.04	+HCOO	451.2830, 441.2997, 197.1183, **149.0465**, 131.0354	15α-Hydroxy-ximicifugoside H_2_^a^	R
111	9.59	C_37_H_60_O_12_	696.4087	696.4085	0.28	−H	487.3424, 469.3302, 425.3438	3-*O*-d-glucopyranosyl platycodigenin methyl ester	S
112	9.80	C_30_H_42_O_7_	514.2938	514.2931	1.41	−H	436.2610, 319.1910, **301.1814**, 265.1468	Marstenacigenin A	R
113	9.91	C_36_H_58_O_12_	682.3893	682.3928	−4.81	+HCOO	**635.3797**, 449.3263, 407.2948, 179.0565	3-*O*-d-glucopyranosyl platycodigenin	R
114	9.94	C_19_H_16_O_7_	356.0886	356.0896	−2.55	+HCOO	401.0868, 313.0718, **121.0297**	6-Formyl-isoophiopogonanone A ^a^	R
115	10.17	C_15_H_18_O_3_	246.1258	246.1256	0.84	+H	**229.1220**, 163.0756, 149.0598, 119.0865, 105.0713	Curcolone ^a^	S, L
116	10.25	C_18_H_34_O_5_	330.2418	330.2406	3.57	−H	**311.2224**, 293.2140, 211.1348, 185.1189, 129.0928	Sanleng acid ^a^	R, S, D
117	10.91	C_15_H_14_O_4_	258.0901	258.0892	3.45	−H	239.0705, 163.0397, **151.0421**, 133.0313, 121.0296	Benzyl-2-hydroxy-6-methoxybenzoate	D
118 *	10.95	C_15_H_20_O_3_	248.1413	248.1412	0.27	+H	231.1379, 219.1381, **203.1425**, 119.0864, 107.0867	Atractylenolide ІІІ	L
119	11.13	C_15_H_20_O_2_	232.1464	232.1463	0.24	+H	215.1424, 187.1486, **159.1172**, 135.1174, 107.0867	Atractylenolide ІІ	S, L
120	12.19	C_16_H_12_O_6_	300.0637	300.0634	1.18	+H	**285.0761**, 242.0571, 167.0340, 136.0162, 108.0215	5-Methyl kaempferol	S, L
121	12.26	C_17_H_14_O_6_	314.0794	314.0790	1.05	+H	299.0552, **275.0673**, 257.0445, 161.0597, 139.0397	3′,5-Dihydroxy-7,4′-dimethoxy flavone	S
122	12.94	C_17_H_26_O_4_	294.1833	294.1831	0.56	−H	235.1341, 141.0919, **129.0924**	6-Gingerol ^a^	R
123	13.46	C_36_H_58_O_12_	682.3905	682.3928	−3.36	−H	**635.3787**, 473.3258, 443.3119, 425.3020, 179.0553	Trachelosperoside B-1 ^a^	D
124	13.68	C_30_H_48_O_5_	488.3514	488.3502	2.47	−H	455.3548, 439.3599, **281.2503**, 293.2127, 171.1035	2*α*,19α-Dihydroxyursolic acid	L
125	13.91	C_18_H_16_O_6_	328.0949	328.0947	0.72	+H	314.0777, **296.0677**, 184.0737, 136.0166	4′,7-Dimethyltectorigenin ^a^	S, L
126 *	14.58	C18H34O4	314.2466	314.2457	2.86	−H	201.1140, 199.0980, 155.1082, 127.1135	Dibutyl sebacate	R
127	14.85	C_19_H_18_O_7_	358.1051	358.1053	−0.47	+H	**343.0809**, 326.0778, 301.0705, 283.0599	3,4-Dihydro-6,8-dihydroxyl-3-(2′-acetyl-3′-hydroxyl-5′-methoxyphenyl)methyl-1*H*-[2] benzoplyran-1-one ^a^	S, L
128	14.86	C_17_H_30_O_2_	266.2258	266.2246	3.76	+HCOO	311.2240, **155.1083**, 139.1137	Methyl 7, 10-hexadecadienoate	R
129	15.36	C_30_H_48_O_7_	520.3385	520.3400	−2.93	−H	476.2774, **473.3256**, 443.3168, 425.3093, 407.2940, 395.2941	Platycodigenin	D
130	15.39	C_17_H_14_O_5_	298.0843	298.0841	0.51	+H	284.0679, **256.0730**, 241.0495, 167.0339, 133.0648	5-Hydroxy-7, 4′-dimethoxyflavanone	S, L
131	15.57	C_26_H_40_O_6_	448.2818	448.2825	−1.59	+H	393.2636, **350.1875**, 242.1877	Tenasogenin ^a^	R
132	15.89	C_14_H_20_O	204.1513	204.1514	−0.51	+H	163.1118, 159.1169, 149.0956, 119.0863, **107.0502**	2-(*p*-Anisyl)-5-methyl-1-hexen	L
133	16.28	C_18_H_16_O_6_	328.0957	328.0947	2.95	+H	**314.0790**, 299.0550, 286.0830, 271.0604, 150.0314	5-Hydro-7, 8, 2′-trimethoxyflavanone	S, L
134	16.57	C_32_H_44_O_9_	572.2965	572.2985	−3.51	−H	**481.2572**, 429.2997, 227.0350, 183.1043	Ganoderic acid H ^a^	L
135	17.23	C_30_H_48_O_4_	472.3550	472.3553	−0.49	−H	471.3448, 437.3061, 419.2937, 339.2705, 253.2187	2α-Hydroxybetulinic acid	S, L
136	17.62	C_16_H_30_O_2_	254.2252	254.2246	2.21	+Na	207.1743, 165.1274, **143.1067**, 125.0961	Palmitoleic acid	R
137	17.78	C_18_H_34_O_3_	298.2505	298.2508	−1.05	−H	217.1615, 195.1391, **183.1401**, 113.0984	Ricinoleic acid	D
138	18.00	C_18_H_30_O_3_	294.2203	294.2195	2.51	+Na	**277.2177**, 165.1284, 151.1127, 109.1035	(*E*,*E*)-9-Oxooctadeca-10,12-dienoic acid ^a^	R
139	18.01	C_18_H_28_O_2_	276.2100	276.2089	3.85	+H	**179.1424**, 135.1180, 119.0862	Stearidonic acid	R
140	18.26	C_28_H_42_N_4_O_6_	530.3100	530.3104	−0.77	−H	529.3027, **511.2928**, 293.2163	Kukoamine A ^a^	R
141	19.02	C_18_H_32_O_3_	296.2358	296.2351	2.19	+Na	279.2312, 161.1323, **147.1165**, 133.1018, 121.1023	Coronaric acid	R
142	19.23	C_28_H_40_O_5_	456.2878	456.2876	0.46	−H	409.2359, 343.1925, **339.2004**, 275.2022	Siraitic acid D ^a^	R
143	20.35	C_32_H_50_O_5_	514.3662	514.3658	0.81	−H	**495.3495**, 469.3702, 451.3596, 449.3449	19α-Hydroxy-3-acetyl-ursolic acid	S
144	20.39	C_30_H_46_O_3_	454.3452	454.3447	1.03	+H	437.3422, **409.3470**, 247.1695, 203.1796, 189.1642	Oleanonic acid	S
145	20.77	C_30_H_48_O_3_	456.3604	456.3603	0.13	−H	455.3531, 443.3528, **233.1561**	3-Epioleanolic acid	S
146	20.78	C_33_H_36_N_4_O_6_	584.2660	584.2635	4.08	+Na	**567.2589**, 535.2340, 501.2257, 467.20432, 417.1830	Bilirubin ^a^	L
147	21.49	C_15_H_30_O	226.2309	226.2297	4.48	+HCOO	**271.2302**, **197.1911**, 195.1754	n-Pentadecanal	S
148	22.20	C_30_H_50_O_2_	442.3803	442.3811	−1.76	+H	425.3776, **407.3666**, 217.1950, 203.1791, 189.1641	Betulin	R
149 *	22.93	C_18_H_30_O_2_	280.2402	280.2400	−0.25	−H	**149.0972**	Linolenic acid	R
150 *	22.95	C_19_H_38_O_4_	330.2774	330.2770	1.00	+Na	313.2738, 239.2368	1-Monopalmitin	S
151	22.98	C_16_H_32_O	240.2452	240.2453	−0.47	+Na	263.2344, 125.1317, **111.1175**	*n*-Hexadecanal	D
152	24.06	C_21_H_42_O	310.3240	310.3236	1.28	+HCOO	355.3214, **125.0972**	*n*-Henicosanal	S
153	24.40	C_16_H_32_O_2_	256.2401	256.2402	−0.49	−H	**241.2176**, 237.226, 227.2019, 125.0976	Palmitic acid	S
154	24.74	C_18_H_34_O_2_	282.2569	282.2559	3.70	−H	253.2185, 163.1132, **125.0982**, 111.0825	Ethyl palmitate	R
155	25.73	C_29_H_46_O	410.3565	410.3549	4.03	+H	**395.3680**, 203.1799, 145.1021, 133.1019	Δ7-stigmasterol	R
156	26.87	C_24_H_38_O_4_	390.2771	390.2770	0.21	+H	301.1413, 189.0156, 165.0905, **149.0235**	Bis(2-ethylhexyl)phthalate	R
157	27.09	C_22_H_43_NO	337.3356	337.3345	3.47	+H	321.3149, **212.2014,** 198.1857, 153.1275	Erucic amide ^a^	R
158	27.63	C_20_H_40_O	296.3093	296.3079	4.10	+HCOO	251.2393, **179.1459**, 113.0987	Phytol	S
159 *	28.49	C_29_H_48_O	412.3695	412.3705	−2.48	+H	**135.1178**, 109.1025	Stigmasterol	R

* Identified with a reference standard. ^a^ Tentatively new identifications in *Campanulaceae.* The fragment ion mass highlighted as bold font is the characteristic MS fragmentation for each compound.

**Table 2:** Information of samples from Jilin Province, China.

Collection Region	Mark of Samples	Collection Date	Collection Region	Mark of Samples	Collection Date
Antu County	S1	2 October 2016	Fusong County	S4	4 October 2016
	L1	2 October 2016		L4	4 October 2016
	R1	26 October 2016		R4	30 October 2016
	D1	2 October 2016		D4	4 October 2016
Hunchun City	S2	1 October 2016	Tonghua City	S5	5 October 2016
	L2	1 October 2016		L5	5 October 2016
	R2	27 October 2016		R5	28 October 2016
	D2	1 October 2016		D5	5 October 2016
Changbai County	S3	30 September 2016	Jiaohe City	S6	3 October 2016
	L3	30 September 2016		L6	3 October 2016
	R3	29 October 2016		R6	25 October 2016
	D3	30 September 2016		D6	3 October 2016

S: stem, L: leaf, R: root; D: seed.
